# Pandemic pressure: policy, politics, profession, and rapid publication

**DOI:** 10.1080/17453674.2020.1753162

**Published:** 2020-04-14

**Authors:** Li Felländer-Tsai

Health care is under siege. Covid-19 has caused a disaster where health care needs by far transcend available resources at the rip curl of the pandemic. Infectious diseases have wreaked havoc before. Lessons have been learnt and sometimes forgotten, despite the sufferings and tragedies of patients and heroic tasks by the healthcare workforce. While the virus is raging, the economy is in free fall with an unprecedented impact on politics and society.

In many ways it has taken the fear of the current pandemic to remind us that many infectious diseases were once so common and deadly, with no choice but to accept the toll. Death by pandemic was dreadful and depressing yet a natural part of life in the world until just some decades ago. But previously polio, malaria, yellow fever, HIV, and cholera have impacted the history of many nations though at a lower speed, apart from, more recently, Ebola and SARS. New dangers have been unleashed by globalization and rapid travel.

Recent and saddening datasets featuring the deaths of medical staff have been published after the initial early reports from China. Not only has ethical and moral stress from having to prioritize scarce resources soared, but also the horror-filled insight that front-line doctors and staff are most at risk. The understanding of the threat that Covid-19 poses to doctors and healthcare staff has initially lagged behind but is now fueling the debate and controversy on state-of-the-art personal protective equipment (PPE) in light of limited supplies. With worries regarding PPE, insurance issues for sick and dead doctors and healthcare staff have also surfaced.

Treatment of severely ill Covid-19 patients has mandated major reorganization of health care, upscaling emergency medicine and trauma while elective surgery is down-prioritized and put on hold. Managing the backlog will create repercussions for a substantial duration. As Covid-19 is spread mainly through airborne transmission, unprotected first-line medical staff and also anesthetists intubating Covid-19 patients are thought to be at particular risk. There is also ongoing debate on the presence of airborne virus transmission in orthopedic surgery caused by power tools and operating room ventilation systems potentially causing viral wind tunnels.

The exit strategies from society lockdowns run in parallel with the global hunt for coronavirus drugs and a vaccine. A promising way of identifying candidate drugs and vaccine candidates is by crunching huge amounts of data and many artificial intelligence companies are now providing their services to scientists. Open sharing of data will be necessary to move forward at speed. And it will be a challenge for pharmaceutical companies to balance commercial versus moral aspects while traversing borders in a collective effort to combat the Covid-19 pandemic.

Rapid media reports and cases of disinformation add to the desperation. Covid-19 has distorted markets, alliances, and policies. Politicians have to seek guidance for decisions while opinions are brokered by an array of experts (including self-appointed ones), government agencies, and non-government organizations in search of stable evidence. World leaders have not always shown signs of cooperation in public-health efforts to fight Covid-19 and the thin line to a blame-game is unfortunately sometimes crossed.

Warnings of the risks of a pandemic have in many instances not been considered when performing risk analyses and planning for supplies and infrastructure. Being in frightening new territory alongside anxiety and fear, it gives solace to remember the huge medical advancements that vaccines and new drugs have enabled over time. During the wait we must acknowledge the selfless contributions by all professionals in medicine, nursing, allied health, research, and also volunteers fighting the war against Covid-19. This is well framed by the transcendent sentence: “*To cure sometimes, to relieve often, to comfort always*.”

Rapid dissemination of new knowledge about coronavirus is important; major scientific journals have introduced fast-track publication of clinical features of the disease, potential drugs and vaccines, effectiveness of preventive measures such as quarantine and even isolation of entire countries. *ACTA* will rapidly electronically publish online relevant orthopedic information related to Covid-19 within an accelerated editorial and review process; please submit material online, category “Corona” (ManuscriptManager).

April 5, 2020

Li Felländer-Tsai, Co-Editor

